# News and Events of the Week

**Published:** 1911-07-15

**Authors:** 


### NEWS AND EVENTS OF THE WEEK.

The first case of smallpox that has been notified for six years in Birmingham was reported last week.

Mr. G. H. Makins, C.B., surgeon to St. Thomas's Hospital, and Mr. C. T. Dent, surgeon to St. George's Hospital, have been elected to the Council of the Royal College of Surgeons.

An annual conference, convened by the National Association for the Prevention of Consumption, is to be held at the Caxton Hall, Westminster, from July 19 to 21. The Tuberculosis Exhibition of the Association will be on view during the week.

The annual health report for the City of Westminster, 1910, by Dr. Allan, the medical officer of health, states that the death rate in the district was 11.0 per 1,000, with an infantile mortality of 78 per 1,000 for legitimate and 129 per 1,000 for illegitimate births, or a total rate of 82 per 1,000. There were 48 cases of typhoid fever notified with seven deaths, a case rate of 2.7 per 10,000 population and a case mortality of 14.5 per cent. According to a report issued by Dr. Hamer to the County Council, it is possible that some of the attacks may be due to the consumption of small plaice or dabs which frequent the more shallow waters, *i.e.* those which would be more liable to pollution. Two cases of epidemic cerebro-spinal meningitis were registered with one death. Under the tuberculosis regulations 275 new cases were notified. The number of deaths from tuberculosis was 231, giving a rate of 135 per 100,000 inhabitants. The number of deaths from phthisis was 186, with a rate of 108 per 100,000 inhabitants. Of non-notifiable diseases, diarrhœa caused the death of 10 per 1,000 infants born.

